# Gut Commensal *Parabacteroides goldsteinii* MTS01 Alters Gut Microbiota Composition and Reduces Cholesterol to Mitigate *Helicobacter pylori*-Induced Pathogenesis

**DOI:** 10.3389/fimmu.2022.916848

**Published:** 2022-06-30

**Authors:** Chih-Ho Lai, Tzu-Lung Lin, Mei-Zi Huang, Shiao-Wen Li, Hui-Yu Wu, Ya-Fang Chiu, Chia-Yu Yang, Cheng-Hsun Chiu, Hsin-Chih Lai

**Affiliations:** ^1^ Department of Microbiology and Immunology, Graduate Institute of Biomedical Sciences, Chang Gung University, Taoyuan, Taiwan; ^2^ Department of Pediatrics, Molecular Infectious Disease Research Center, Chang Gung Memorial Hospital at Linkou, Taoyuan, Taiwan; ^3^ Department of Microbiology, School of Medicine, China Medical University, Taichung, Taiwan; ^4^ Department of Nursing, Asia University, Taichung, Taiwan; ^5^ Department of Medical Biotechnology and Laboratory Science, College of Medicine, Chang Gung University, Taoyuan, Taiwan; ^6^ Microbiota Research Center and Emerging Viral Infections Research Center, Chang Gung University, Taoyuan, Taiwan; ^7^ Molecular Medicine Research Center, Chang Gung University, Taoyuan, Taiwan; ^8^ Department of Laboratory Medicine, Chang Gung Memorial Hospital at Linkou, Taoyuan, Taiwan; ^9^ Department of Otolaryngology-Head and Neck Surgery, Chang Gung Memorial Hospital, Taoyuan, Taiwan; ^10^ Medical Research Center, Xiamen Chang Gung hospital, Xiamen, China; ^11^ Research Center for Chinese Herbal Medicine, College of Human Ecology, Chang Gung University of Science and Technology, Taoyuan, Taiwan

**Keywords:** *Parabacteroides goldsteinii*, gut microbiota, *Helicobacter pylori*, cholesterol, pathogenesis

## Abstract

*Helicobacter pylori* infection is closely associated with various gastrointestinal diseases and poses a serious threat to human health owing to its increasing antimicrobial resistance. *H. pylori* possesses two major virulence factors, vacuolating cytotoxin A (VacA) and cytotoxin-associated gene A (CagA), which are involved in its pathogenesis. Probiotics have recently been used to eradicate *H. pylori* infection and reduce the adverse effects of antibiotic-based therapies. *Parabacteroides goldsteinii* MTS01 is a novel next-generation probiotic (NGP) with activities that can alleviate specific diseases by altering the gut microbiota. However, the mechanism by which *P. goldsteinii* MTS01 exerts its probiotic effects against *H. pylori* infection remains unclear. Our results showed that administration of *P. goldsteinii* MTS01 to *H. pylori*-infected model mice altered the composition of the gut microbiota and significantly reduced serum cholesterol levels, which mitigated *H. pylori*-induced gastric inflammation. In addition, the pathogenic effects of *H. pylori* VacA and CagA on gastric epithelial cells were markedly abrogated by treatment with *P. goldsteinii* MTS01. These results indicate that *P. goldsteinii* MTS01 can modulate gut microbiota composition and has anti-virulence factor functions, and thus could be developed as a novel functional probiotic for reducing *H. pylori*-induced pathogenesis.

## Introduction

Persistent *Helicobacter pylori* infection in the human stomach is closely associated with the development of various gastrointestinal diseases, including chronic gastritis, peptic ulcers, and gastric cancer (1). Combination therapies using several antibiotics and either a proton pump inhibitor (PPI) or bismuth are widely employed to eradicate *H. pylori* and relieve related gastrointestinal illnesses (2). However, a consequent increase in antibiotic resistance and changes in the gut microbiota often occur (3, 4).

The two main virulence factors of *H. pylori*, vacuolating cytotoxin A (VacA) and cytotoxin-associated gene A (CagA), are involved in its pathogenesis (5). VacA is a pore-forming toxin that promotes acidic vacuole formation in host cells and inhibits mitochondrial function, resulting in apoptosis (6). CagA, which is encoded on the *cag*-pathogenicity island (*cag*-PAI), is delivered to host cells through the type IV secretion system (TFSS) and activates nuclear factor-kappa B (NF-κB) and IL-8 secretion, leading to inflammation and, in some cases, oncogenesis (7). Notably, these two virulence factors exploit cholesterol-rich microdomains in membranes, which are referred to as lipid rafts, for cell binding, intracellular delivery, and cell intoxication (8, 9). Therefore, depleting cholesterol to disrupt lipid rafts is considered an ideal strategy for alleviating *H. pylori*-related diseases (10).

Probiotics and their metabolites play crucial roles in promoting host health, including maintaining physiological homeostasis, improving intestinal integrity, and producing antimicrobial peptides (11). Recently, several traditional probiotics were used to eradicate *H. pylori* infection and reduce the adverse effects of antibiotic-based therapies (2). However, the effectiveness of probiotics for eradicating *H. pylori* is controversial (12, 13). The conflicts regarding the clinical effectiveness of traditional probiotics could be resolved by next-generation probiotics (NGP), which have fully defined genetic backgrounds and physiological characteristics that target specific diseases (14).

*Parabacteroides goldsteinii* MTS01, a newly discovered NGP, exerts multiple disease-alleviating effects, including obesity-reversing, insulin resistance-controlling, and COPD pathogenesis-ameliorating effects, by altering the gut microbiota (15–17). A recent study reported that a loss of *P. goldsteinii* may exacerbate colitis in model mice (18). However, whether *P. goldsteinii* MTS01 can alter this microbial ecosystem and mitigate *H. pylori*-induced pathogenesis in the stomach remains to be explored. In this study, we comprehensively investigated the mechanisms by which *P. goldsteinii* MTS01 inhibits *H. pylori* infection and ameliorates inflammation in the stomach using a mouse model. Our results showed that administration of *P. goldsteinii* MTS01 to the model mice altered the composition of the gut microbiota, reduced serum cholesterol, and effectively attenuated the actions of *H. pylori* VacA and CagA. These findings suggest that *P. goldsteinii* MTS01 could be developed as a functional probiotic against *H. pylori*-associated pathogenesis.

## Materials and Methods

### Bacterial Culture

*H. pylori* strain 26695 (ATCC 700392) was cultured on the blood agar plates (Brucella agar with 10% defibrinated sheep blood) and incubated at 37°C in microaerophilic environment (5% O_2_, 10% CO_2_, and 85% N_2_). *P. goldsteinii* MTS01 was isolated from the feces of a healthy adult who has taken *P. goldsteinii* MTS01 previously isolated from a healthy mouse (15) for half a year. It was cultured in thioglycollate medium (BD Biosciences) at 37°C in an anaerobic chamber as described previously (15). Culture supernatant from *P. goldsteinii* MTS01 was filtrated using a 0.22 μm filter. Heat-inactivated *P. goldsteinii* MTS01 was prepared by boiling 1×10^8^ CFU/ml of bacterial suspension in PBS for 30 min. Live, heat-inactivated, and culture broth of *P. goldsteinii* MTS01 were collected for following analyses.

### Animal Experiments

**Figure 1:**
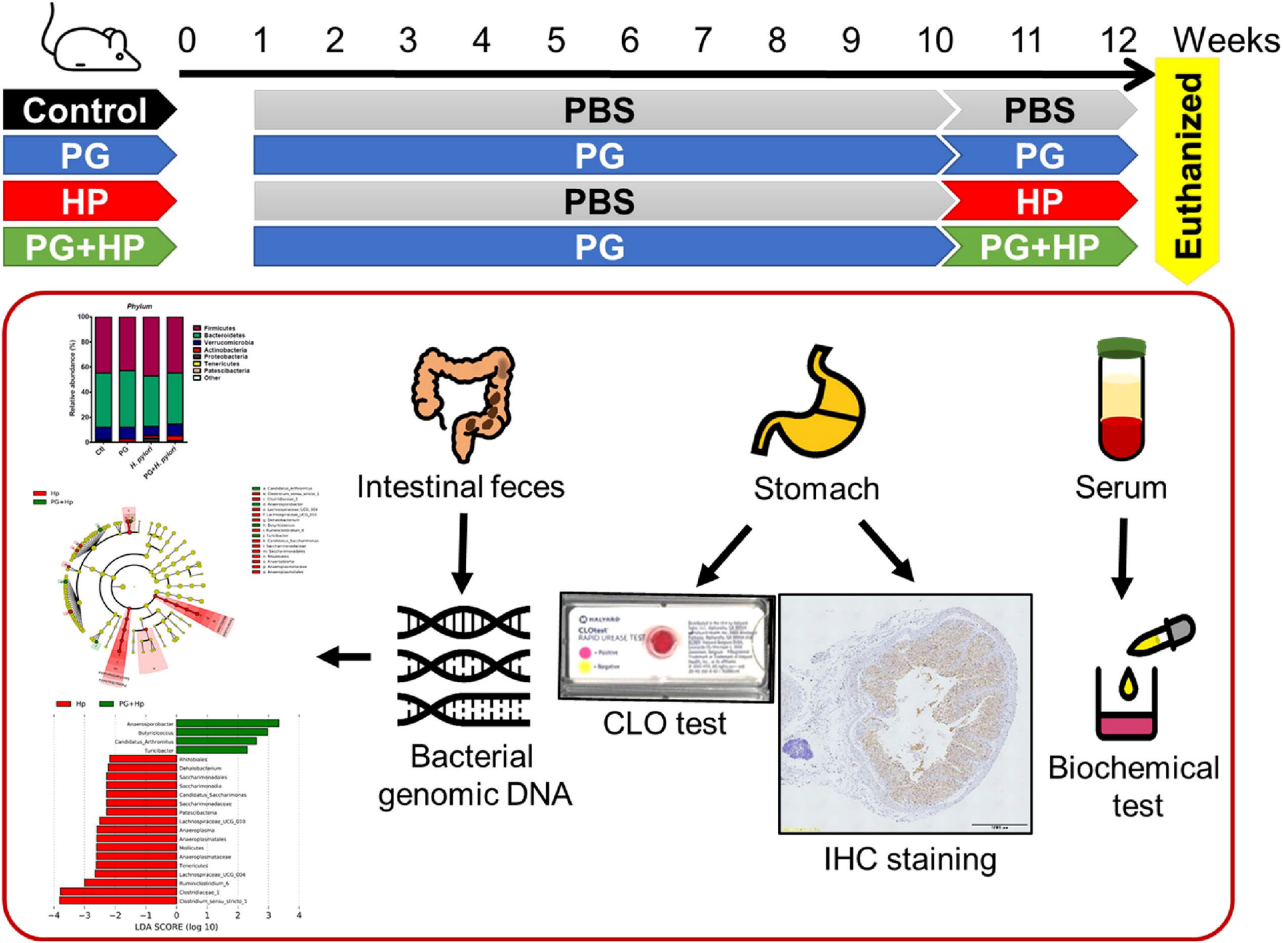
Murine model for experimental studies. Mice were divided into four groups for the treatments with vehicle-control (PBS, n = 9), *P. goldsteinii* MTS01 (PG, n = 10), *H. pylori* (HP, n = 8), and *P. goldsteinii* MTS01 + *H. pylori* (n = 10). Mice were intragastrically administered *P. goldsteinii* MTS01 (2×10^8^ CFU/100 μl) once daily for a total of nine weeks and continuously inoculated with *P. goldsteinii* MTS01 and *H. pylori* (1×10^9^ CFU/100 μl) once daily for additional two weeks. Mice were euthanized and the samples of blood, stomach, and stool were prepared for further analysis.

Six-week-old male BALB/c mice were purchased from the National Laboratory Animal Center (Taipei, Taiwan). The mouse experiments were performed in accordance with the Animal Care and Use Guidelines for Chang Gung University under a protocol approved by the Institutional Animal Care Use Committee (IACUC Approval No.: CGU107-141). The body weight and rectal temperature were recorded every morning during the study period (**Supplementary Figure 1**). Mice were randomly divided into 4 groups for the treatments with vehicle control (PBS) (n = 9), *P. goldsteinii* MTS01 (n = 10), *H. pylori* (n = 8), and *P. goldsteinii* MTS01 + *H. pylori* (n = 10), respectively (**Figure 1**). Mice were fed with *P. goldsteinii* MTS01 (2×10^8^ CFU/100 μl) by intragastric gavage once daily for a total of 9 weeks. Mice were then inoculated with *H. pylori* (1×10^9^ CFU/100 μl) once every two days and continuously fed with *P. goldsteinii* MTS01 (2×10^8^ CFU/100 μl) once daily for additional two weeks. After completing the treatment course, mice were euthanized and the blood samples, gastric tissues, and intestinal stool were prepared for further analysis, as described previously (19).

### Serum Cholesterol/Triglyceride Analysis

Mouse blood was collected by cardiac puncture and centrifuged immediately (7000 rpm for 10 min) to prepare the serum. Serum triglyceride was analyzed using a Triglyceride Assay Kit (ab65336, Abcam). The levels of total cholesterol, high-density lipoprotein (HDL), low-density lipoprotein (LDL), and very-low-density lipoproteins (VLDL) in serum were assayed by a Cholesterol Assay ELISA Kit (ab65390, Abcam).

### Rapid Urease Test

CLO test (Kimberly Clark), a rapid urease test used to determine *H. pylori* infection. A gastric biopsy from mice was added into the CLO test microtube and incubated at room temperature for 3 h. The change in color of the gastric biopsies from yellow to red, which indicated the positive colonization of *H. pylori* (20).

### Histopathological Analysis

Murine gastric tissues were prepared for immunohistochemistry (IHC) staining as described previously (16). The tissue sections were stained with monoclonal antibodies (Abcam) against COX-2 (1:1000), IL-1β (1:100), and TNF-α (1:100) at 4°C for 18 h. After washing, the tissue sections were incubated with a horseradish peroxidase (HRP)-conjugated goat anti-rabbit secondary antibody (1:000, GBI Labs) and developed with an ABC kit (Vector Laboratories). The stained tissues were analyzed by a histopathologist using a light microscope (Carl Zeiss).

### Gut Microbiota Analysis

Gut or fecal microbiota containing both intestinal and gastric microbiota may affect the pathology of *H. pylori* infection (21, 22), and we analyzed their composition. Microbiota DNA in mouse stool was extracted using the QIAamp PowerFecal Pro DNA Kit (Qiagen) and applied to the amplification of 16S rRNA V3–V4 region using PCR, as described previously (23, 24). In brief, 16S amplicon libraries were constructed by two-step PCR reactions and the concentrations of each library was assessed using an HT DNA High Sensitivity LabChip kit (Caliper). The multiplexed amplified libraries were sequenced using the Illumina MiSeq system. The amplicon sequences were analyzed by following the MiSeq SOP (25). As a result, a total of 986 amplicon sequence variants (ASVs) was obtained, then ASVs were annotated into taxonomic orders using the get_taxa_unique function in phyloseq (26). The species richness among the gut microbiota was determined using the alpha diversity measures: observed species richness, Shannon, and Fisher’s diversity index by phyloseq. Two-dimensional PCoA plots were used to analyze the bacterial community populations between two groups using phyloseq. Beta diversity was conducted by using weighted Unifrac phylogenetic distance matrices (27). A PERMANOVA (α = 0.05) with 999 random permutations was run to determine differences between groups with the Vegan package. The cladogram functionality of the linear discriminant analysis (LDA) effect size (LEfSe) method was used to illustrate differences in gut microbiota composition between un-treatment and treatment with *P. goldsteinii* MTS01. In the cladogram, differences between groups were illustrated at phyla, class, order, family, and genus levels. PICRUSt was used to predict the microbial functionality profiles by generating the Kyoto Encyclopedia of Genes and Genomes (KEGG) pathway according to 16S rRNA gene sequencing (28). The predicted genes were aligned to KEGG database and the different pathways among groups were compared using STAMP (version 2.1.3) with two-side Welch’s t-test.

### Cell Culture

AGS cells (ATCC CRL-1739, human gastric epithelial cell line) were cultured in F12 medium (Hyclone) containing 10% fetal bovine serum (Hyclone) and incubated at 37°C in an incubator with 5% CO_2_.

### Determination of Cell Elongated Phenotype

AGS cells were pretreated with *P. goldsteinii* MTS01 (MOI = 200) followed by infection with *H. pylori* (MOI = 100) for 6 h. *H. pylori* VacA-induced cell vacuolation and CagA-induced elongated cells (hummingbird phenotype) were observed. The percentage of elongated cells was determined as reported previously (29).

### CagA Phosphorylation Assay

AGS cells were pretreated with *P. goldsteinii* MTS01 (MOI 200) for 30 min followed by infection with *H. pylori* (MOI = 100) for additional 6 h. The cell extracts were prepared and subjected to 6% SDS-PAGE then transferred onto polyvinylidene difluoride membranes (1:1000, Millipore). The membranes were incubated with anti-phosphotyrosine (4G10) antibody (1:2000, Millipore) overnight at 4°C, and then probed with a horseradish peroxidase-conjugated secondary antibody (Millipore). The expression level of phospho-CagA was analyzed using ECL western blotting detection reagents (GE Healthcare) and recorded by AzureSpot Analysis Software with Azure 400 (Azure Biosystems).

### NF-κB Luciferase Activity Assay

AGS cells were transfected with NF-κB-luciferase reporter followed by treatment with *P. goldsteinii* MTS01 (MOI = 200) and *H. pylori* (MOI = 100) for 6 h. The cell lysates were prepared and subjected to luciferase assays using Dual-Luciferase Reporter Assay System (Promega). Luciferase activity was normalized by co-transfection of β-galactosidase expression vector (Promega), as described previously (29).

### Analysis of IL-8 Production

Culture supernatant from AGS cells was collected. The IL-8 level was analyzed using enzyme-linked immunosorbent (ELISA) assay according to the manufacturer’s instructions (Invitrogen) (30).

### Statistical Analysis

Differential abundance was analyzed using the Kruskal-Wallis to detect main effect differences and then applied to Wilcoxon rank-sum test for pairwise comparisons (24). Mann–Whitney *U* test was used to evaluate differences in microbiota abundance (31). In the biochemistry and cell-based studies, statistical significance was determined by Student’s *t*-test between two groups. One-way ANOVA test with Tukey *post-hoc* test was used to assess the statistical significance of the experimental results for more than two studied groups. The statistical analysis was conducted using SPSS program (version 18.0 for Windows, SPSS Inc.) and the figures were performed by Prism program (version 9.0.0 for Windows, GraphPad Software). A *P* value less than 0.05 was considered statistically significant.

## Results

### *P. goldsteinii* MTS01 Influences the Gut Microbiota Diversity

To assess the effects of *P. goldsteinii* MTS01 on *H. pylori*-induced gastric inflammation *in vivo*, mice were inoculated with live *P. goldsteinii* MTS01 *via* intragastric gavage before *H. pylori* infection. Mice were divided into the following four groups: control (PBS), *P. goldsteinii* MTS01 (PG), *H. pylori* (HP), and PG+HP (**Figure 1**). After treatment, the mice were euthanized, and intestinal feces were collected to characterize the gut microbiota using 16S rRNA V3–V4 sequencing. The average number of raw reads per sample was 198,893 for the 37 specimens tested (**Supplementary Table 1**). A total of 4,767,390 high-quality filtered reads (mean, 128,848.4 ± 23,599.6 reads per sample) were obtained. The reads were constructed into 986 ASVs, and these sequence variants were used for taxonomic classification. The ASVs were then categorized into 8 phyla, 13 classes, 19 orders, 29 families, and 75 genera. The taxonomic and phylogenetic data for ASVs are shown in **Supplementary Table 2**.

**Figure 2:**
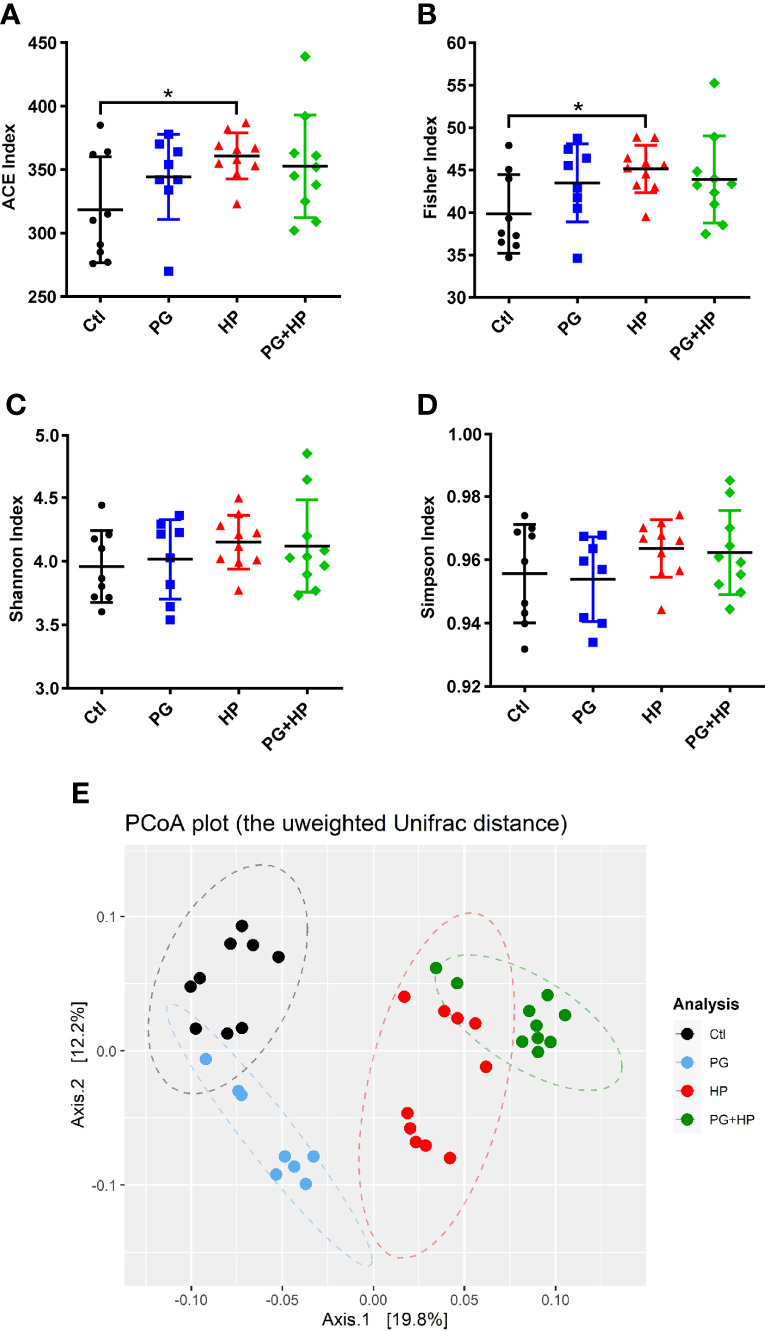
*P. goldsteinii* MTS01 and *H pylori* alter the gut microbiota communities. Diversity index of **(A)** ACE, **(B)** Fisher, **(C)** Shannon, and **(D)** Simpson were performed to analyze microbiota diversity in murine intestinal stool. *P < 0.05 was assessed using Student’s *t*-test. **(E)** Analysis of the gut microbiota diversity. PCoA was conducted to analyze the gut microbiota composition in mice administered with vehicle-control (black), *P. goldsteinii* MTS01 (blue), *H. pylori* (red), and *P. goldsteinii* MTS01 + *H. pylori* (green).

We then compared the composition and diversity of the microbiota in mice subjected to the different treatments. Compared to the control group, the gut microbiota communities of *H. pylori*-infected mice had significantly higher richness indices, including the abundance-based coverage estimator (ACE)-diversity index and Fisher’s index (**Figures 2A, B**). In contrast, the microbiota communities in *P. goldsteinii* MTS01-treated and *H. pylori*-infected (PG+HP) mice were less diverse than those in *H. pylori*-infected (HP) mice, as shown by the four alpha diversity indices (**Figures 2A–D**). Principal coordinate analysis (PCoA) was used to examine the beta diversity of the four treatment groups. Our results showed that the spatial separation of the gut microbiota composition in the PCoA plot varied among the different treatments (Adonis test, *P*=0.001 and R^2 =^ 0.37; **Figure 2E**). These results indicated that the gut microbiota profiles differed among the four treatment groups.

### *P. goldsteinii* MTS01 Alters Relative Abundance of Gut Microbiota

**Figure 3:**
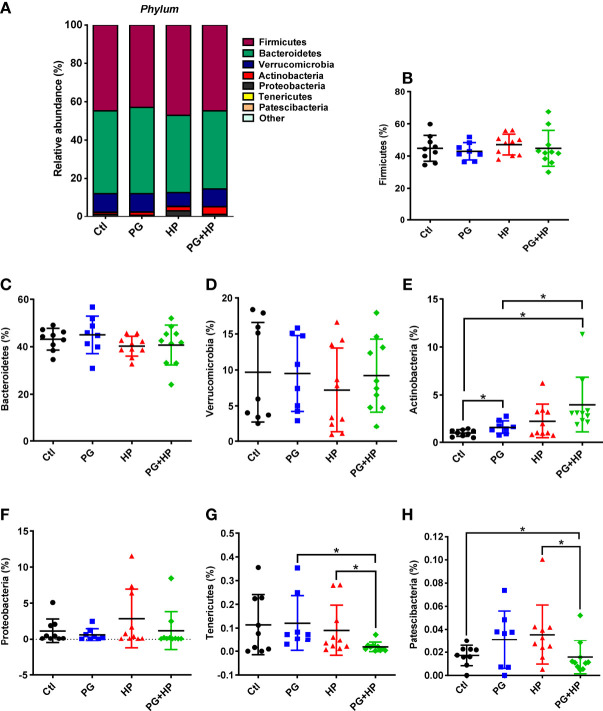
Relative abundance of the gut microbiota in phyla. The bacteria taxonomic profiles at phylum in the gut microbiota in *P. goldsteinii* MTS01-treated and *H. pylori*-infected mice. **(A)** Top seven most abundant phyla in the four murine groups were analyzed. Relative abundance of **(B)** Firmicutes, **(C)** Bacteroidetes, **(D)** Verrucomicrobia, **(E)** Actinobacteria, **(F)** Proteobacteria, **(G)** Tenericutes, and **(H)** Patescibacteria in the gut microbiota community were assessed. *P < 0.05.

Next, we investigated the profiles of the major microbiota at phylum and genus levels in the four treatment groups. In the control group, the top seven most abundant phyla were Firmicutes (44.8 ± 8.1%), Bacteroidetes (43.2 ± 4.6%), Verrucomicrobia (9.7 ± 6.9%), Actinobacteria (0.98 ± 0.4%), Proteobacteria (1.1 ± 1.7%), Tenericutes (0.1 ± 0.1%), and Patescibacteria (0.02 ± 0.01%; **Figure 3).** There were no significant differences in the levels of Firmicutes, Bacteroidetes, Verrucomicrobia, and Proteobacteria among the four treatment groups (**Figures 3B−D, F**). However, in the *H. pylori*-infected group, the abundance of *Firmicutes* (47.1%, *P*=0.45) increased, while the abundance of *Bacteroidetes* (40.2%, *P*=0.18) decreased compared to their abundances in control mice, but these differences were not significant (**Figure 3B, C**). Notably, the Firmicutes/Bacteroidetes ratio was lower in *P. goldsteinii* MTS01-fed *H. pylori*-infected mice than that in *H. pylori*-infected mice. Moreover, Tenericutes (0.02%, *P*=0.028) and Patescibacteria (0.02%, *P*=0.029) were significantly decreased in *P. goldsteinii* MTS01-fed *H. pylori*-infected mice compared to their levels in *H. pylori*-infected mice (**Figure 3G, H**).

**Figure 4:**
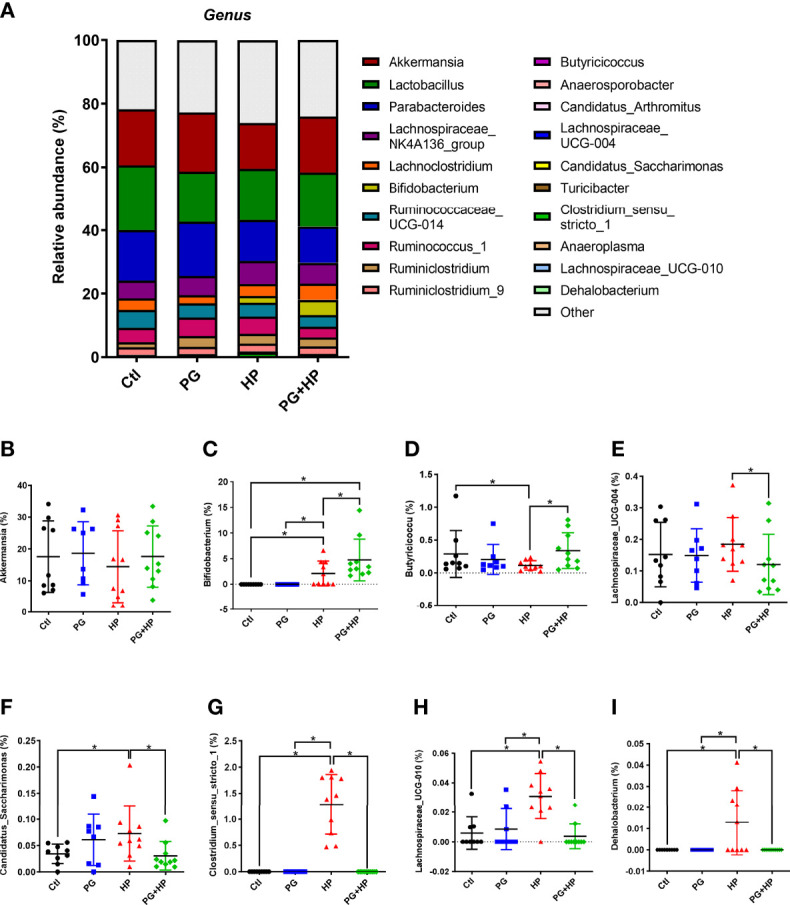
Relative abundance of the gut microbiota in genera. The bacteria taxonomic profiles at genus in the gut microbiota in mice administrated with *P. goldsteinii* MTS01 and *H. pylori*. **(A)** Top twenty most abundant genera among four treated groups were shown. Relative abundance of **(B)** Akkermansia, **(C)** Bifidobacterium, **(D)** Butyricicoccus, **(E)** Lachnospiraceae_*UCG-004*, **(F)** Candidatus_Saccharimonas, **(G)** Clostridium_sensu_stricto_1, **(H)** Lachnospiraceae_UCG-010, **(I)** Dehalobacterium in the gut microbiota were analyzed. *P < 0.05.

The most abundant bacteria were then analyzed at the genus level, and the top 20 genera were identified (frequency > 0.01%; **Figure 4** and **Supplementary Figure 2**). Compared to the control group, seven genera were significantly changed in the *H. pylori* infection group; six genera were increased (Bifidobacterium, Candidatus*_*Saccharimonas, Clostridium*_*sensu*_*stricto*_*1, Lachnospiraceae_UCG-010, Dehalobacterium, and Ruminiclostridium), and one genus (Butyricicoccus) was decreased (*P* < 0.05). Comparison of PG+HP and HP mice showed that eleven genera were different; five genera were increased (Bifidobacterium, Butyricicoccus, Anaerosporobacter, Candidatus*_*Arthromitus, and Turicibacter), and six genera were decreased (Lachnospiraceae_UCG-004, Candidatus*_*Saccharimonas, Clostridium*_*sensu*_*stricto*_*1, Lachnospiraceae_UCG-010, Dehalobacterium, and Anaeroplasma). These results indicate that the gut bacterial community was modulated in mice exposed to either *P. goldsteinii* MTS01 and/or *H. pylori*.

### *P. goldsteinii* MTS01 Changes the Genera of Gut Microbiota

**Figure 5:**
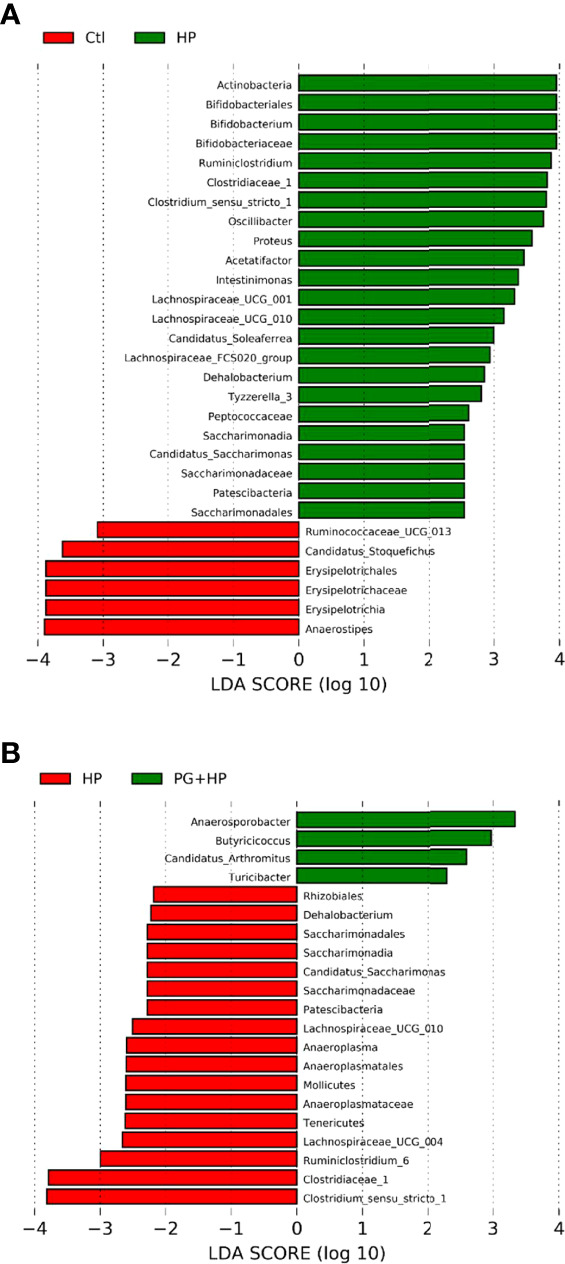
Differences in the gut microbiota composition in *P. goldsteinii* MTS01-treated and *H pylori*-infected mice. LEfSe analysis exhibited the abundance bacterial species compared with **(A)** control and *H. pylori*; **(B)** *H. pylori* and *P. goldsteinii* MTS01+*H. pylori*.

**Figure 6:**
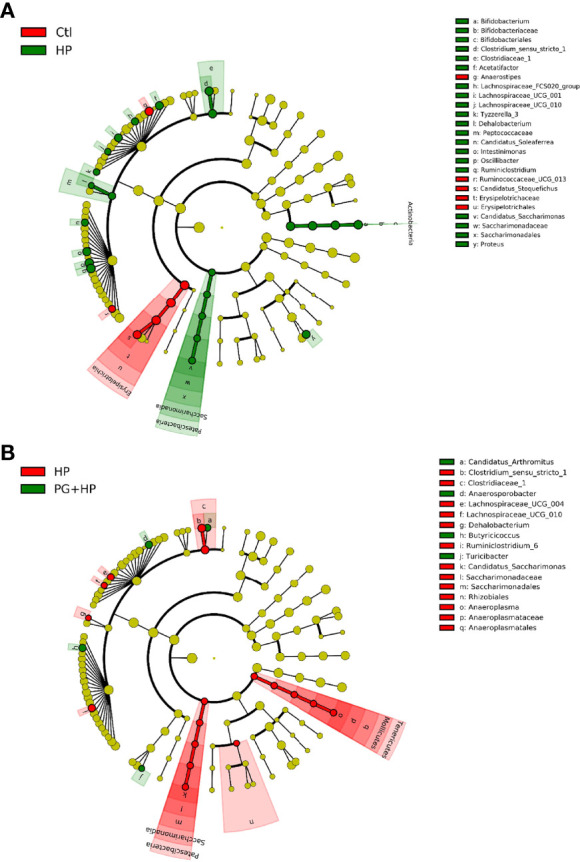
Circular taxonomic and phylogenetic tree of the gut microbiota diversity. Compared the effect of *P. goldsteinii* MTS01 and *H. pylori* altered microbiota composition in each group. Cladogram showed enriched taxa of gut microbiome in mice treated with **(A)** control and *H. pylori*; **(B)** *H. pylori* and *P. goldsteinii* MTS01+*H. pylori*.

We next analyzed the relative abundance of bacteria at the genus level among the experimental groups using LEfSe analysis (**Figure 5** and **Suplementary Figure 3**). Our results showed that 17 genera were altered between the control and HP groups and 10 genera were altered between the HP and PG+HP groups. The abundances of the top six enriched bacterial genera, *Actinobacteria*, *Bifidobacterium*, *Ruminiclostridium*, *Oscillibacter*, *Proteus*, and *Intestinimonas*, were significantly more abundant in *H. pylori*-infected mice than in control mice (**Figure 5A**, **Supplementary Table 3**). The relative abundances of *Anaerosporobacter*, *Butyricicoccus*, *Candidatus_Arthromitus*, and *Turicibacter* were significantly higher in the PG+HP group than in the HP group (**Figure 5B**). We generated a cladogram of the circular taxonomic and phylogenetic relationships based on the relative abundance data, which identified the bacterial genera that were differentially present in the HP and PG+HP groups (**Figure 6** and **Supplementary Figure 4**). These results indicate that the gut microbiota of *H. pylori*-infected mice was influenced by treatment with *P. goldsteinii* MTS01.

### Functional Analysis of the Bacterial Communities

**Figure 7:**
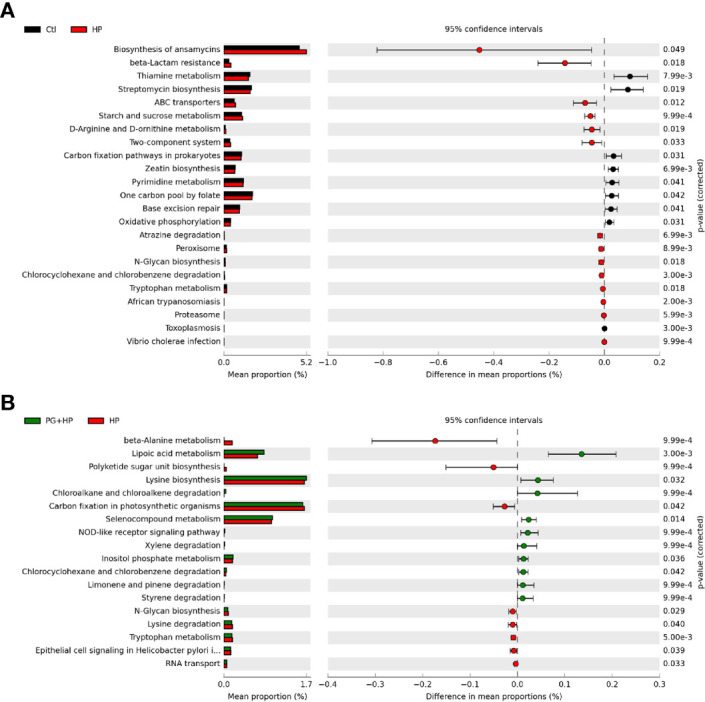
Putative functions of microbiota community. The microbial functionality profiles altered by *P. goldsteinii* MTS01 and *H. pylori* were analyzed using PICRUSt2 to generate KEGG pathway. Functional features showed the comparison between **(A)** control and *H. pylori*, and **(B)** *H. pylori* and *P. goldsteinii* MTS01+*H. pylori*.

We used PICRUSt2 to analyze the differences in microbiome function resulting from *P. goldsteinii* MTS01 treatment in *H. pylori*-infected mice (**Figure 7** and **Supplementary Figure 5**). The major pathways correlated with *H. pylori* infection were thiamine metabolism, two-component system, and N-glycan biosynthesis (**Figure 7**). The predominant pathways that were altered following *P. goldsteinii* MTS01 treatment in *H. pylori*-infected mice were lipoic acid metabolism, lysine biosynthesis, NOD-like receptor signaling pathway, and inositol phosphate metabolism. These findings indicate that the differentially abundant bacteria in mice administered *P. goldsteinii* MTS01 were associated with lipid metabolism and the host immune response, which may contribute to the amelioration of *H. pylori*-induced pathogenesis.

### *P. goldsteinii* MTS01 Reduces Serum Cholesterol Level and Mitigates Gastric Inflammation in *H. pylori*-Infected Mice

**Figure 8:**
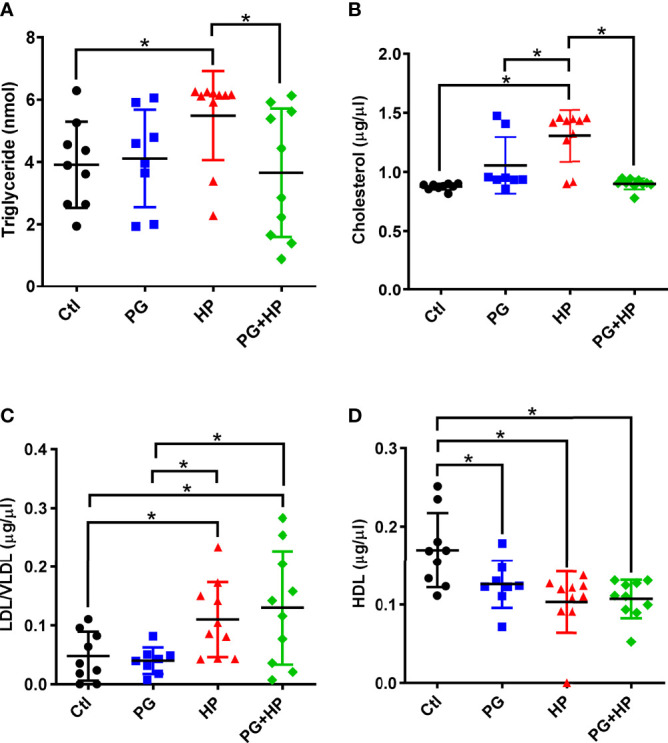
*P. goldsteinii* MTS01 lowers serum triglyceride/cholesterol. Sera from mice were collected and the levels of **(A)** triglyceride, **(B)** total cholesterol, **(C)** LDL/VLDL, and **(D)** HDL were analyzed. *P < 0.05.

**Figure 9:**
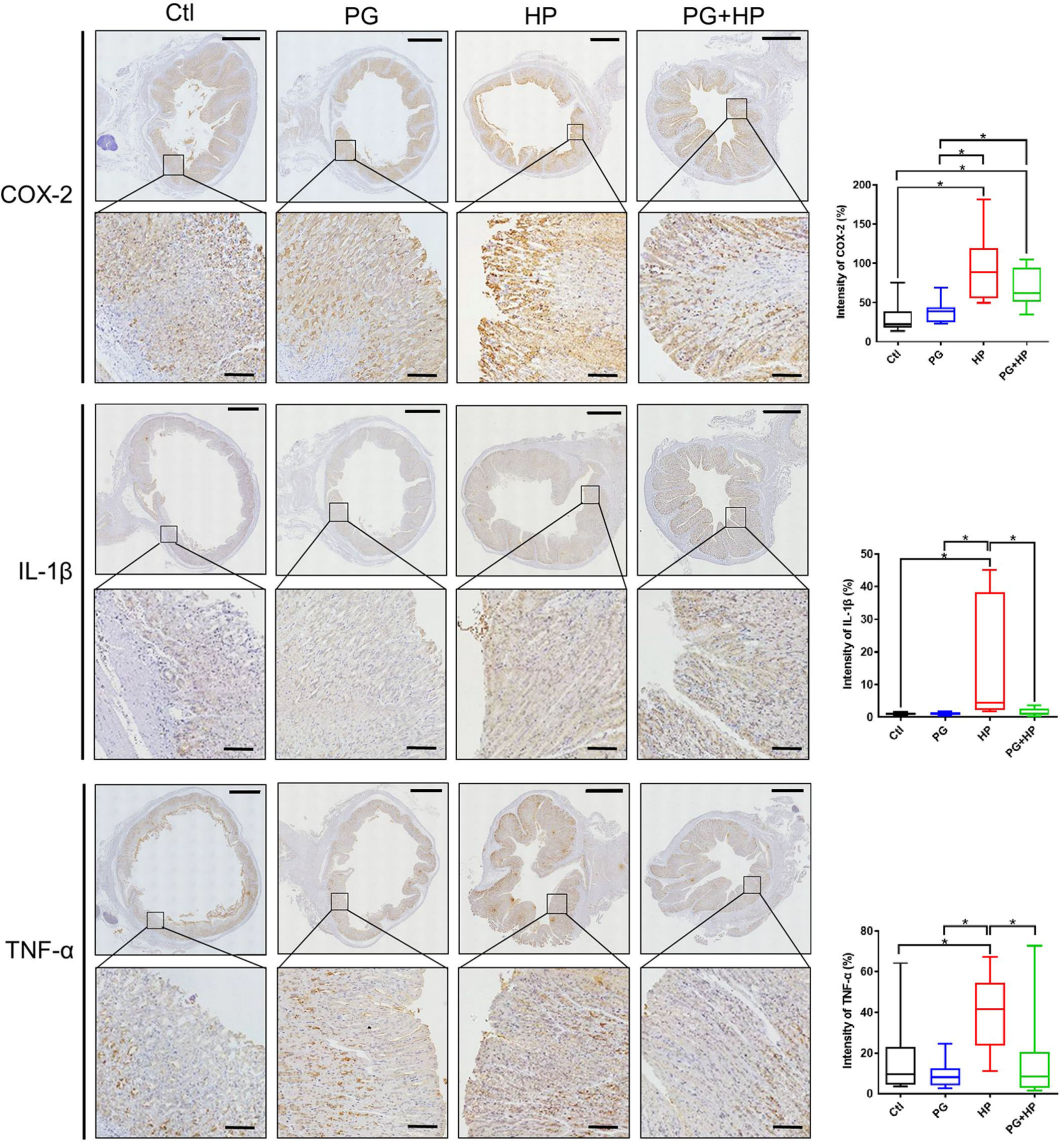
*P. goldsteinii* MTS01 mitigates *H. pylori*-induced gastric inflammation. Mouse gastric tissues were subjected to IHC staining with specific antibodies against COX-2, IL-1β, and TNF-α (original magnification: 200×). The magnified images are displayed below each cropped area. Scale in each panel, 1000 μm, and in each magnified image, 100 μm. The intensity of COX-2, IL-1β, and TNF-α expression for IHC staining in gastric tissues are shown in the right panels. "*" indicated P < 0.05.

To further investigate whether *P. goldsteinii* MTS01 alleviates *H. pylori*-induced pathogenesis mediated by reducing cholesterol levels, we analyzed mouse serum triglyceride/cholesterol levels and prepared gastric tissues for histopathological analysis. Our results showed that serum levels of triglycerides and total cholesterol were significantly increased in mice infected with *H. pylori* compared to those in the control mice (**Figures 8A, B**). *P. goldsteinii* MTS01 treatment effectively inhibited these increases in serum triglyceride and cholesterol levels in *H. pylori*-infected mice, but did not affect LDL/VLDL and HDL levels when compared to those in *H. pylori*-infected mice. Mouse gastric tissues were subjected to histopathological analysis. IHC analysis showed faint expression of pro-inflammatory cytokines (COX-2, IL-1β, and TNF-α) in the gastric tissues of control mice, indicating an absence of inflammation (**Figure 9**). In contrast, the expression of these proinflammatory cytokines was much higher in *H. pylori*-infected tissues but was remarkably reduced in *H. pylori*-infected mice fed *P. goldsteinii* MTS01. However, the CLO test indicated that *H. pylori* infection in the stomach was unaffected by *P. goldsteinii* MTS01 treatment (data not shown). Together, these results demonstrate that *P. goldsteinii* MTS01 treatment decreased serum cholesterol, which is associated with the amelioration of stomach inflammation caused by *H. pylori*.

### *P. goldsteinii* MTS01 Alleviates *H. pylori*-Induced Pathogenesis

**Figure 10:**
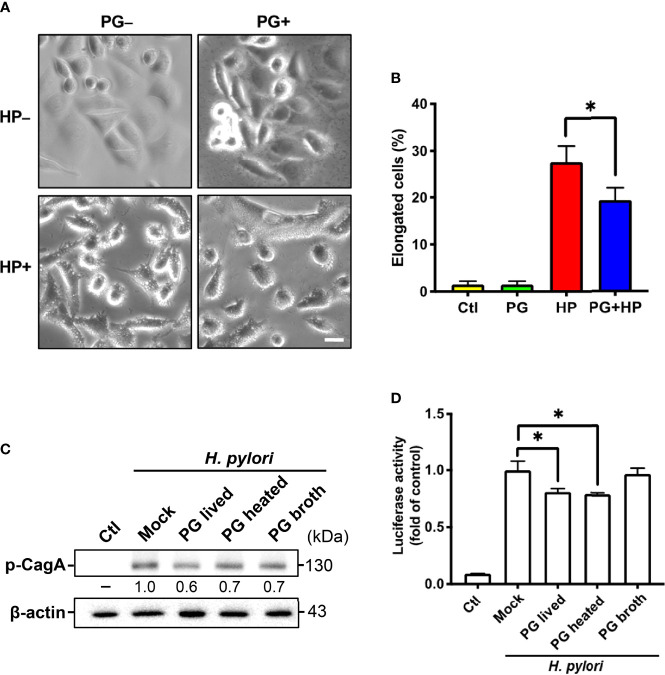
*P. goldsteinii* MTS01 ameliorates *H. pylori*-induced pathogenesis. **(A)** AGS cells were pretreated with *P. goldsteinii* MTS01 for 30 min and then infected with *H. pylori* at an MOI of 100 for 6 h. Cell vacuolation and elongation were observed by using a phase-contrast microscope. **(B)** The proportion of elongated cells were counted. **(C)** CagA phosphorylation were analyzed by western blot assay. Relative protein expression levels were normalized to β-actin and indicated under each lane. **(D)** The level of NF-κB activation was assessed using luciferase assay. *P < 0.05.

We investigated whether *P. goldsteinii* MTS01 lowers cholesterol levels and consequently attenuates VacA and CagA activity in cells. AGS cells were pretreated with *P. goldsteinii* MTS01 for 30 min and infected with *H. pylori* for 6 h. As shown in **Figure 10A**, *H. pylori* VacA-induced vacuole formation was effectively diminished in cells pretreated with *P. goldsteinii* MTS01. Similarly, the *H. pylori* CagA-induced scattering (hummingbird) phenotype was significantly decreased in *P. goldsteinii* MTS01-pretreated cells (**Figure 10B**). We then investigated whether *P. goldsteinii* MTS01 inhibited *H. pylori* CagA phosphorylation in cells. Our results showed that phosphorylation of CagA was increased in *H. pylori*-infected cells, but was significantly decreased in cells pretreated with either live or heat-inactivated *P. goldsteinii* MTS01 (**Figure 10C**). This trend was also observed for *H. pylori*-induced NF-κB activation (**Figure 10D**). Collectively, our results demonstrate that *P. goldsteinii* MTS01 inhibits the pathogenic effects of *H. pylori* VacA and CagA in gastric epithelial cells, which may be attributed to its microbiota-altering and cholesterol-lowering effects.

## Discussion

Several bacteria use virulence factors that hijack cellular cholesterol to gain access to host cells (32, 33). Two virulence factors of *H. pylori*, VacA and CagA, exploit cholesterol for cellular internalization and consequent disease development (8, 9). Lowering cellular cholesterol has been shown to effectively abrogate the progression of diseases induced by pathogenic bacteria (34). For instance, cholesterol-lowering agents, such as statins, have been used to inhibit bacterial infectivity (35–37). In our recent studies, we found that statin therapy reduced serum cholesterol levels and decreased the incidence of *H. pylori*-related diseases, indicating that cholesterol plays a pivotal role in the function of *H. pylori* virulence factors (38–40). In the present study, we showed that *P. goldsteinii* MTS01 has potent serum triglyceride/cholesterol-lowering activity, which is associated with reduced *H. pylori*-induced gastric inflammation in mouse models. In addition, treatment of cells with *P. goldsteinii* MTS01 remarkably inhibited the effects of VacA and CagA, which in turn decreased NF-κB activation and proinflammatory cytokine production. In our previous study, we showed that administration of live *P. goldsteinii* MTS01 reversed obesity in HFD-fed mice, mitigated endotoxemia, and relieved inflammation (15). Given these beneficial effects, *P. goldsteinii* MTS01 could be developed into a beneficial probiotic to combat *H. pylori* virulence factors and alleviate related inflammation and disease.

Eradication therapy using a combination of antibiotics and PPI is the gold standard for the treatment of *H. pylori* infections (2). However, with the widespread use of antibiotics, antimicrobial resistance of *H. pylori* has increased significantly, increasing the incidence of treatment failure (41). In addition, antibiotics can alter the gut microbiota and cause dysbiosis, which adversely affects physiological functions and may exacerbate disease progression (42). However, co-treatment of patients with both antibiotics and probiotics has been shown to prevent the adverse effects associated with antibiotic use and increase the rates of *H. pylori* eradication (43–45). This effect can be explained by the fact that probiotic supplements improve the dysbiosis caused by antibiotic eradication therapy (46). Consistent with previous reports, the results of the current study indicate that *P. goldsteinii* MTS01 has the potential to be developed as a functional probiotic for ameliorating *H. pylori*-induced pathogenesis.

An increased ratio of Firmicutes/Bacteroidetes was associated with *H. pylori*-positive gastritis (47) and obesity (48), and a reversal of the increased Firmicutes/Bacteroidetes ratio has been shown to be associated with reductions in total cholesterol, obesity, and related inflammation (17, 49, 50). Our results showed an increase in *Bifidobacterium* and a decrease in *Clostridium* in *H. pylori*-infected mice administered *P. goldsteinii* MTS01. In addition, *P. goldsteinii* MTS01 treatment decreased the Firmicutes/Bacteroidetes ratio, demonstrating that *P. goldsteinii* MTS01 could help maintain gut normobiosis and restore the disruption in the intestinal microbiota caused by *H. pylori* infection.

*H. pylori* CagA co-localized with the tight junction scaffolding protein ZO-1 at sites of bacterial attachment, resulting in the disruption of epithelial barrier function (51). Destruction of tight junctions by CagA resulted in a wider cell gap and altered cell-cell contact, which in turn enhanced cell migration (52). Consequently, sequelae may occur, particularly peptic ulcer disease and gastric cancer. Disruption of gastrointestinal barrier integrity caused leakage of intestinal bacterial LPS and proinflammatory cytokines into the circulation, leading to exacerbation of endotoxemia and inflammation (53). The present results show that *P. goldsteinii* MTS01 has inhibitory effects against *H. pylori*-CagA functions, including CagA phosphorylation and cell morphology scattering. We recently reported that *P. goldsteinii* MTS01 treatment effectively alleviated LPS-induced monolayer disruption and restored ZO-1 expression, which are crucial for maintaining gut integrity and homeostasis (15). Collectively, these results indicate that *P. goldsteinii* MTS01 possesses both antibacterial virulence factor effects and gut barrier-maintaining functions.

LPS, which is produced by gram-negative bacteria, is recognized by TLR4 and activates MD-2 to trigger signaling in response to inflammation (54). LPS derived from *H. pylori* is involved in the activation of the TLR4/MD-2 signalling pathway, which induces bacterial-elicited inflammation in gastric epithelial cells (55, 56). Notably, we recently reported that *P. goldsteinii* MTS01-LPS antagonizes *E. coli* LPS-induced activation, and the effect was attributed to competition for TLR4 (16). This is similar to the findings with LPS isolated from *Bacteroides* and *Rhodobacter* spp., which possess potent antagonism against pathogenic LPS-induced inflammation (57–59). Since the present study demonstrated the inhibitory effects of *P. goldsteinii* MTS01 against *H. pylori*-associated pathogenesis, whether the mechanism underlying the alleviation of H. pylori-induced inflammation is mediated by P. goldsteinii *MTS01*-LPS deserves further investigation.

In conclusion, we employed murine models to investigate the potential inhibitory effects of *P. goldsteinii* MTS01 on *H. pylori* infection and its physiological effects *in vivo* and to elucidate the detailed mechanism. Our results showed that *P. goldsteinii* MTS01 lowered cholesterol levels, significantly decreased the effects of *H. pylori* virulence factors on cells, and diminished bacteria-induced inflammation. *P. goldsteinii* MTS01 also altered the microbiota composition; increasing the number of beneficial bacteria and maintaining gut microbiota normobiosis. Our results demonstrate that *P. goldsteinii* MTS01 could be developed as a novel NGP to target *H. pylori* infection and alleviate its pathogenesis.

## Data Availability Statement

The data presented in the study are deposited in the NCBI repository, accession number PRJNA833917.

## Ethics Statement 

The animal study was reviewed and approved by the Institutional Animal Care Use Committee of Chang Gung University (IACUC Approval No.: CGU107-141).

## Author Contributions

C-HL, C-HC, and H-CL designed this work. T-LL, M-ZH, H-YW, and Y-FC performed the experimental study. T-LL, M-ZH, H-YW, and Y-FC, S-WL, and C-YY performed data analysis and interpretation. C-HL, T-LL, C-YY, C-HC, and H-CL. wrote the manuscript. All authors reviewed and approved the manuscript.

## Conflict of Interest

The authors declare that the research was conducted in the absence of any commercial or financial relationships that could be construed as a potential conflict of interest.

## Publisher’s Note

All claims expressed in this article are solely those of the authors and do not necessarily represent those of their affiliated organizations, or those of the publisher, the editors and the reviewers. Any product that may be evaluated in this article, or claim that may be made by its manufacturer, is not guaranteed or endorsed by the publisher.

## Supplementary Material

Supplementary Figure 1 Body weight and temperature of mice during experimental studies.Mice were divided into four groups for the treatments with vehicle-control (PBS, n = 9), *P. goldsteinii* MTS01 (PG, n = 10), *H. pylori* (HP, n = 8), and *P. goldsteinii* MTS01 + *H. pylori* (n = 10). The body weight and temperature of the mice were recorded every week for a total of twelve weeks.

Supplementary Figure 2 Relative abundance of the gut microbiota in genera. The bacteria taxonomic profiles at genus level in the gut microbiota of the experimental mice. Relative abundance of **(A)** Lactobacillus, **(B)** Parabacteroides, **(C)** Lachnospiraceae_NK4A136_group, **(D)** Lachnoclostridium, **(E)** Ruminococcus_UCG014, **(F)** Ruminococcus_1, **(G)** Ruminiclostridium, **(H)** Ruminiclostridium_9, **(I)** Anaerosporobacter, **(J)** Candidatus_Arthromitus, **(K)** Turicibacter, **(L)** Anaeroplasma in the gut microbiota were analyzed. **P* < 0.05.

Supplementary Figure 3 Differences in gut microbiota composition in mice administrated with P. goldsteinii MTS01 and H. pylori. LEfSe analysis exhibited the abundance bacterial species compared with **(A)** control and *P. goldsteinii* MTS01+*H. pylori*; **(B)** *H. pylori* and *P. goldsteinii* MTS01.

Supplementary Figure 4 Circular taxonomic and phylogenetic tree of gut microbiota diversity. Compared the effect of *P. goldsteinii* MTS01 and *H. pylori* altered microbiota composition in each group. Cladogram showed enriched taxa of gut microbiome in mice treated with **(A)** control and *P. goldsteinii* MTS01+*H. pylori*; **(B)** *H. pylori* and *P. goldsteinii* MTS01.

Supplementary Figure 5 Putative functions of microbiota community. The microbial functionality profiles altered by *P. goldsteinii* MTS01 and *H. pylori* were analyzed using PICRUSt2 to generate KEGG pathway. Functional features showed the comparison between **(A)** control and *P. goldsteinii* MTS01+*H. pylori*; **(B)** *H. pylori* and *P. goldsteinii* MTS01.
