# Supplementary figures and images for: Gut Commensal Parabacteroides goldsteinii MTS01 Alters Gut Microbiota Composition and Reduces Cholesterol to Mitigate Helicobacter pylori-Induced Pathogenesis
Source: Front Immunol. 2022 Jun 30;13:916848. doi: 10.3389/fimmu.2022.916848 (PMC9281563; doi:10.3389/fimmu.2022.916848)

Fig. S1

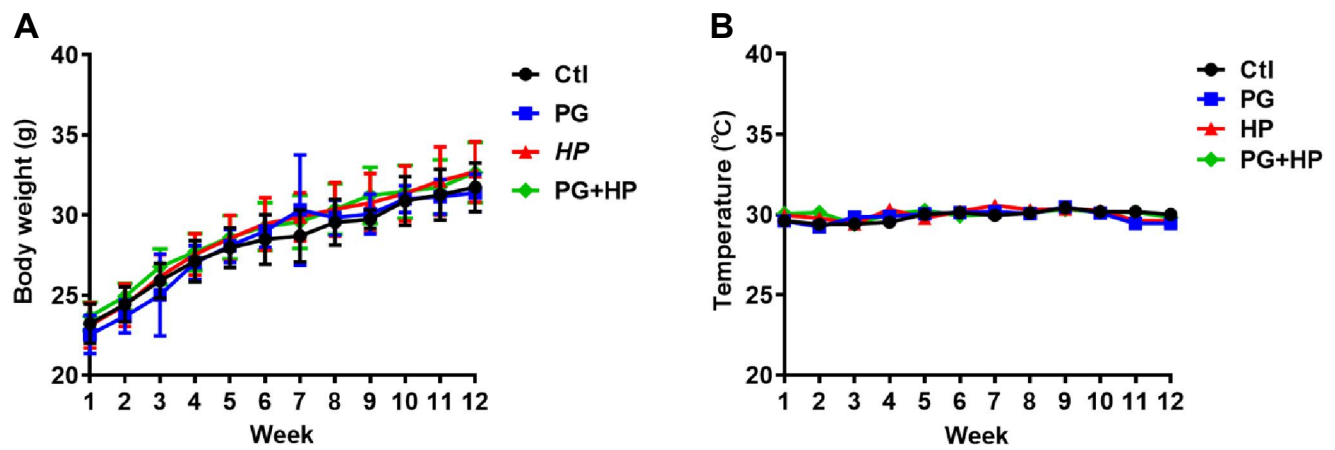

Supplement: Supplementary Figure 1 — Body weight and temperature of mice during experimental studies.Mice were divided into four groups for the treatments with vehicle-control (PBS, n = 9), P. goldsteinii MTS01 (PG, n = 10), H. pylori (HP, n = 8), and P. goldsteinii MTS01 + H. pylori (n = 10). The body weight and temperature of the mice were recorded every week for a total of twelve weeks. [file Image_1.pdf]

Fig. S2

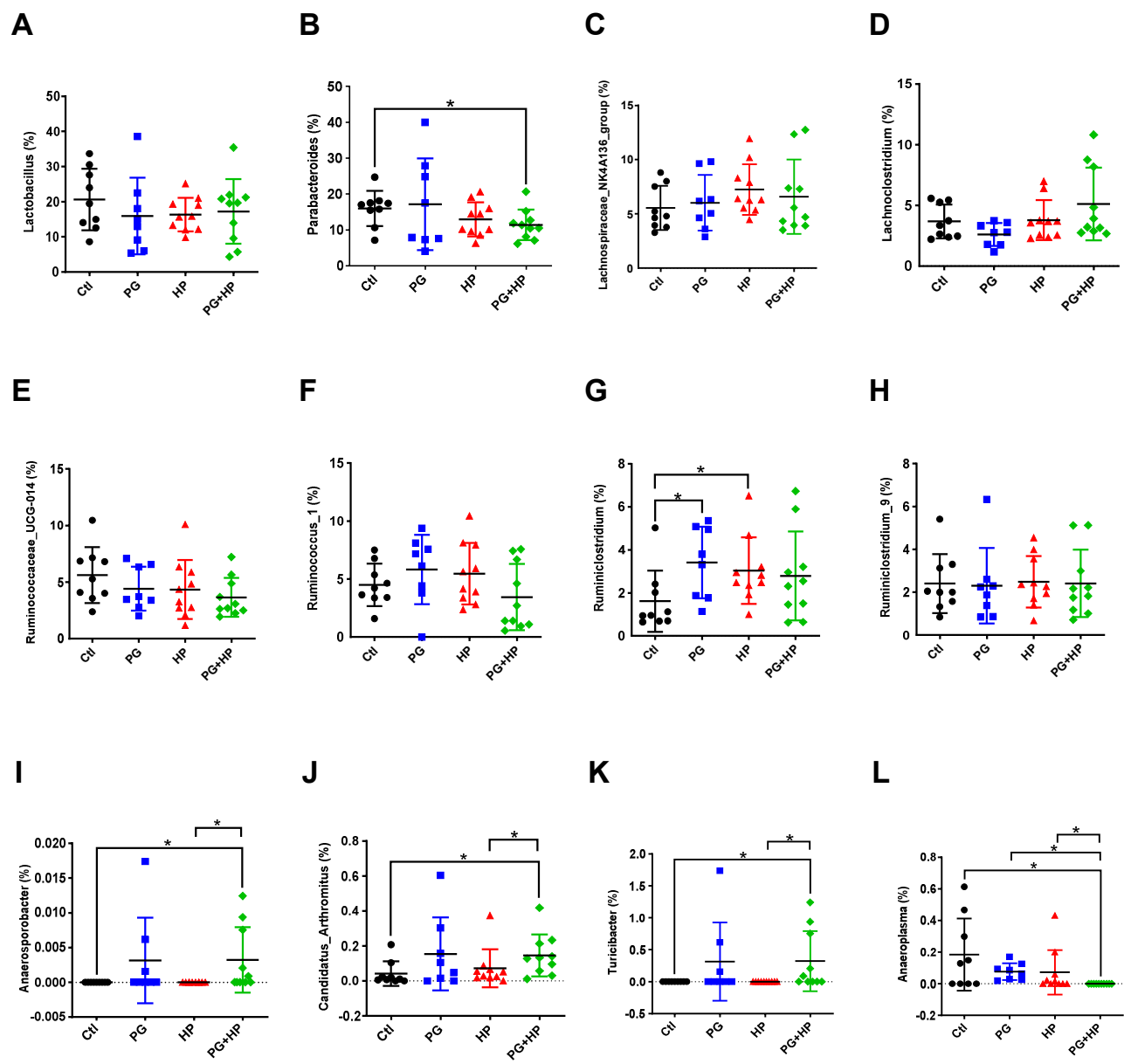

Supplement: Supplementary Figure 2 — Relative abundance of the gut microbiota in genera. The bacteria taxonomic profiles at genus level in the gut microbiota of the experimental mice. Relative abundance of (A) Lactobacillus, (B) Parabacteroides, (C) Lachnospiraceae_NK4A136_group, (D) Lachnoclostridium, (E) Ruminococcus_UCG014, (F) Ruminococcus_1, (G) Ruminiclostridium, (H) Ruminiclostridium_9, (I) Anaerosporobacter, (J) Candidatus_Arthromitus, (K) Turicibacter, (L) Anaeroplasma in the gut microbiota were analyzed. *P < 0.05. [file Image_2.pdf]

Fig. S3

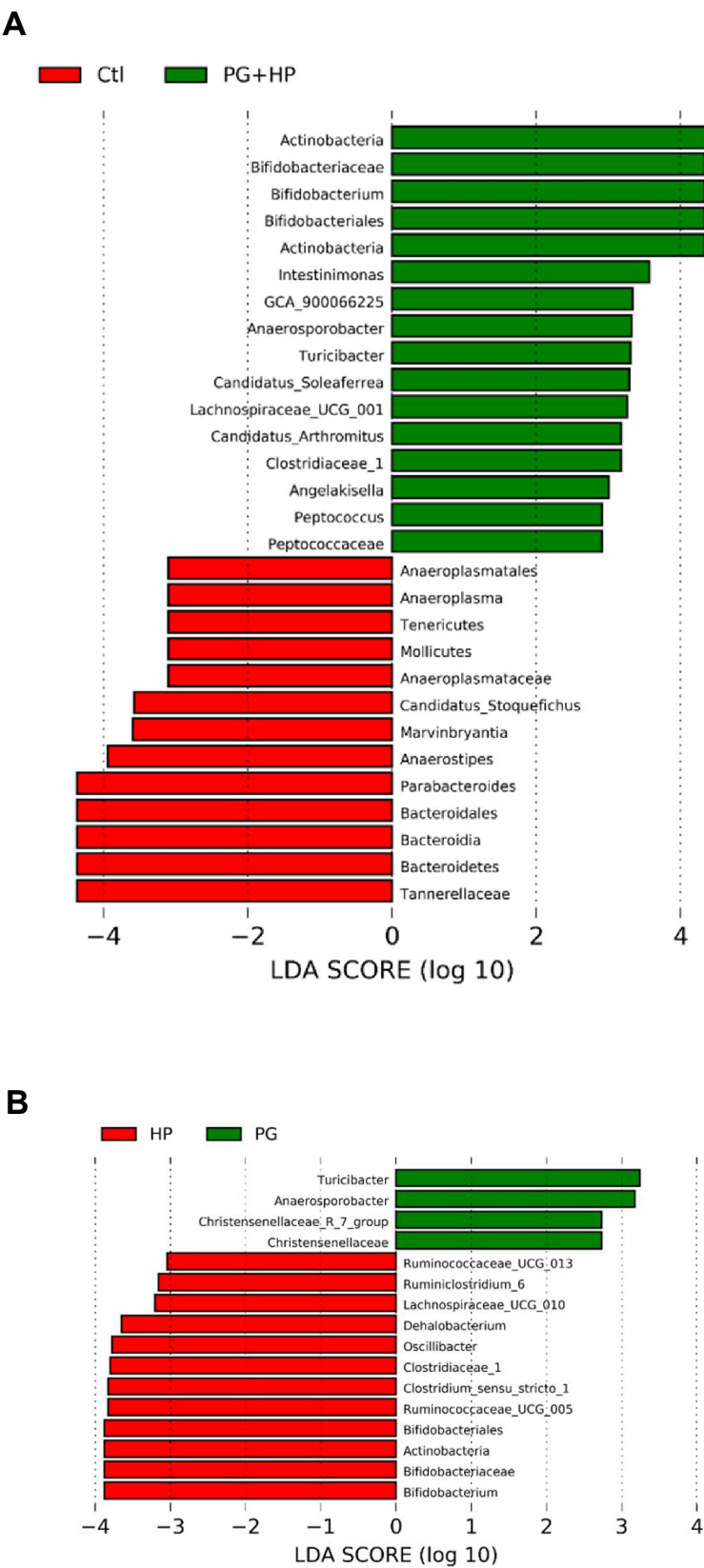

Supplement: Supplementary Figure 3 — Differences in gut microbiota composition in mice administrated with P. goldsteinii MTS01 and H. pylori. LEfSe analysis exhibited the abundance bacterial species compared with (A) control and P. goldsteinii MTS01+H. pylori; (B) H. pylori and P. goldsteinii MTS01. [file Image_3.pdf]

Fig. S4

A

Ctl  
PG+HP

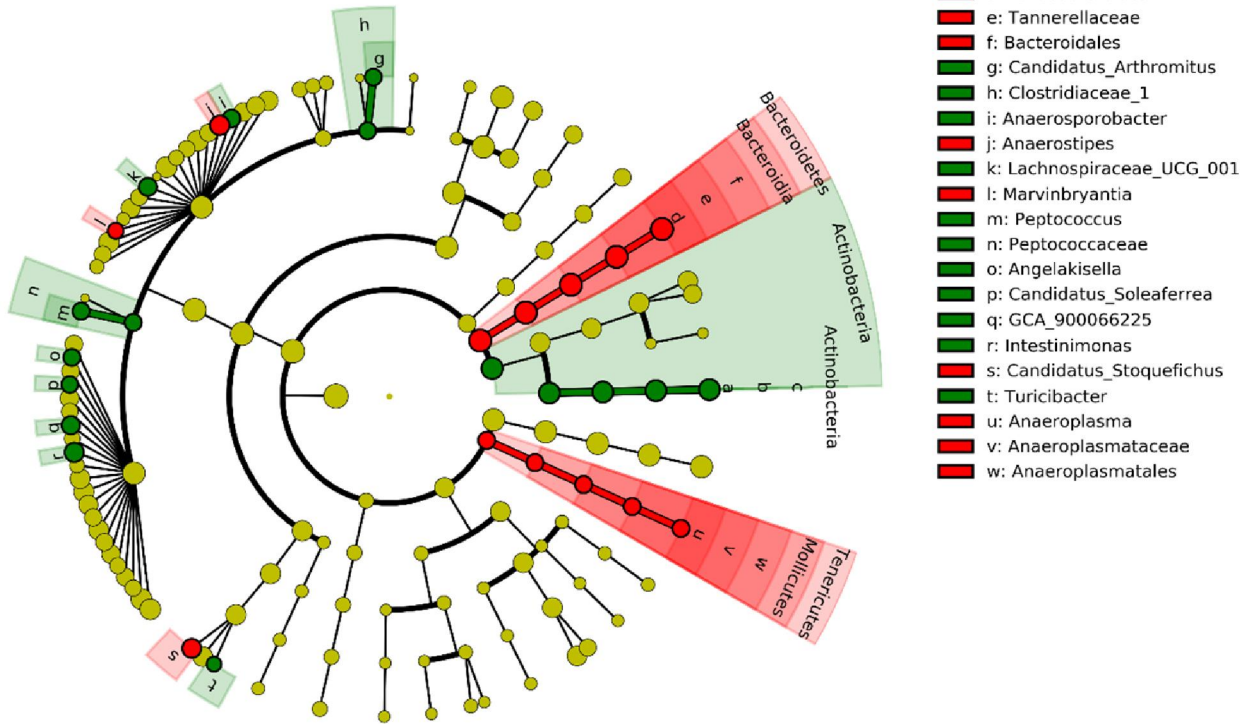

B

HP  
PG

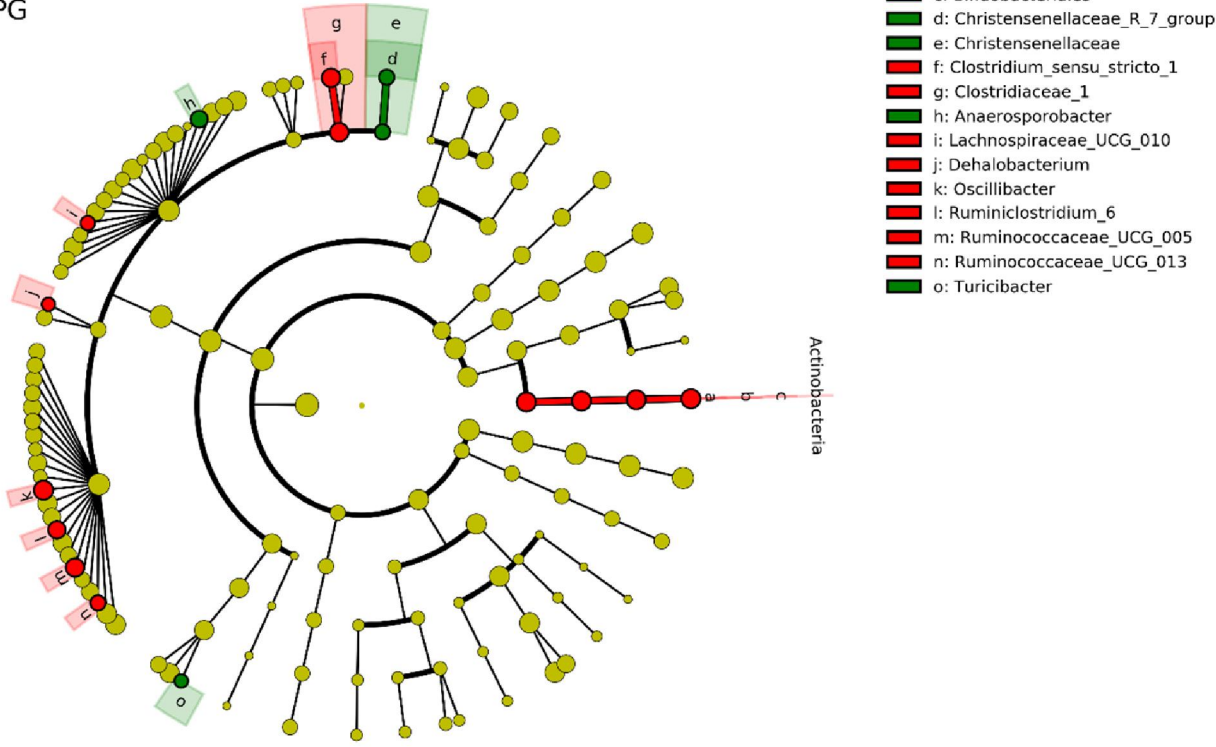

Supplement: Supplementary Figure 4 — Circular taxonomic and phylogenetic tree of gut microbiota diversity. Compared the effect of P. goldsteinii MTS01 and H. pylori altered microbiota composition in each group. Cladogram showed enriched taxa of gut microbiome in mice treated with (A) control and P. goldsteinii MTS01+H. pylori; (B) H. pylori and P. goldsteinii MTS01. [file Image_4.pdf]

Fig. S5

A

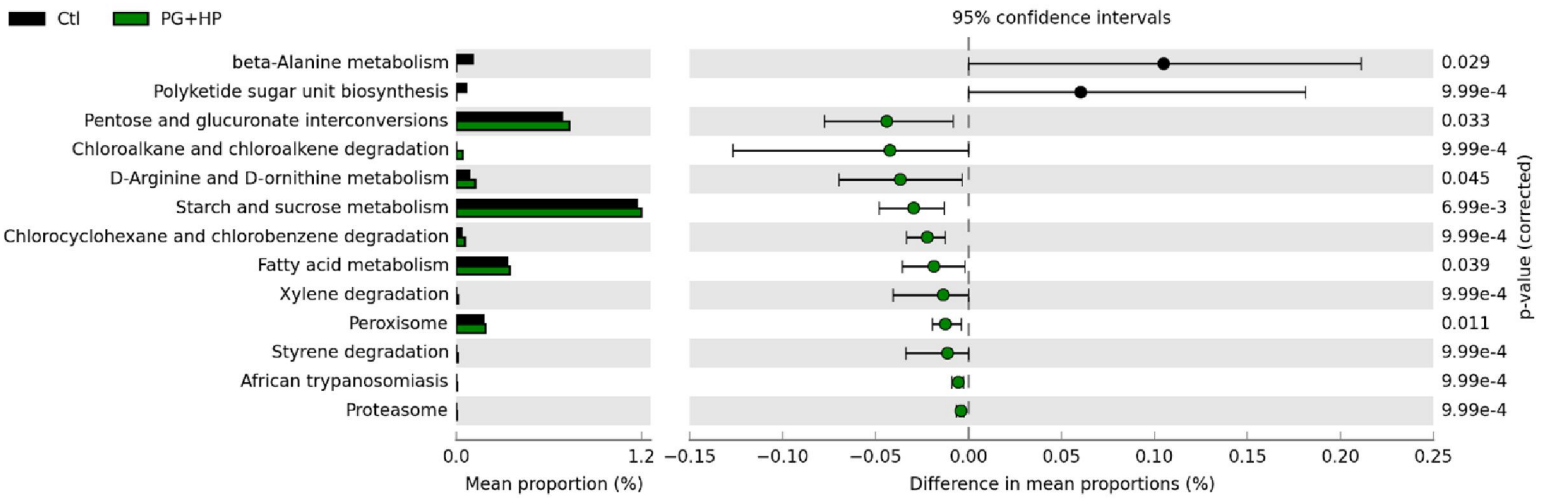

B

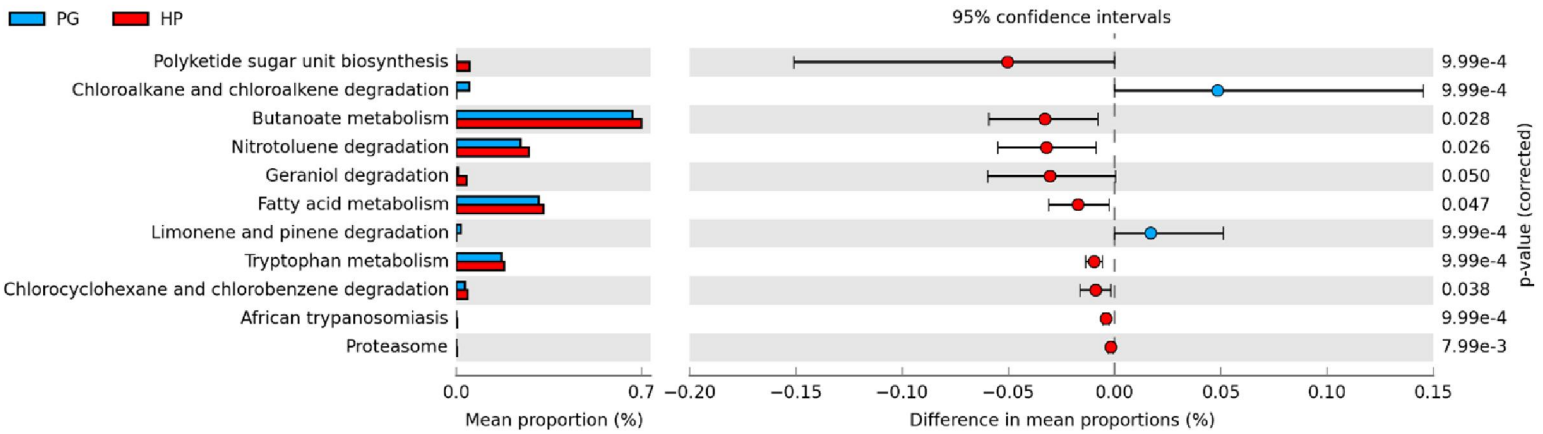

Supplement: Supplementary Figure 5 — Putative functions of microbiota community. The microbial functionality profiles altered by P. goldsteinii MTS01 and H. pylori were analyzed using PICRUSt2 to generate KEGG pathway. Functional features showed the comparison between (A) control and P. goldsteinii MTS01+H. pylori; (B) H. pylori and P. goldsteinii MTS01. [file Image_5.pdf]
